# What are the similarities and differences in lung cancer symptom appraisal and help‐seeking according to smoking status? A qualitative study with lung cancer patients

**DOI:** 10.1002/pon.6041

**Published:** 2022-11-25

**Authors:** Georgia B. Black, Sandra van Os, Katriina L. Whitaker, Gill S. Hawkins, Samantha L. Quaife

**Affiliations:** ^1^ Department of Applied Health Research University College London London UK; ^2^ Centre for Prevention Detection and Diagnosis Wolfson Institute of Population Health Barts and The London School of Medicine and Dentistry Queen Mary University of London London UK; ^3^ School of Health Sciences University of Surrey Guildford UK

**Keywords:** diagnostic uncertainty, help‐seeking, lung cancer, patient safety, risk appraisal, smoking history

## Abstract

**Objective:**

Lung cancer in never‐smokers represents a growing proportion of patients. The relationship between smoking status, symptom appraisal and help‐seeking behaviour is complex. Little is known about cancer symptom‐related health behaviours according to smoking status. The aim of the study was to explore lung cancer patients' experiences of a lung cancer diagnosis, identifying differences by smoking history.

**Method:**

This was a qualitative study involving telephone interviews with 40 lung cancer patients (20 never smokers, 11 former smokers and 9 current smokers). We used framework analysis to analyse the data using the Common Sense Model of Illness Self‐Regulation as a theoretical framework, developed after initial analysis.

**Results:**

All patients were likely to delay seeking help for symptoms in primary care regardless of smoking history, but for different reasons. Smoking history was instrumental to how individuals perceived and responded to early symptoms of lung cancer. Differences in interpretation and coping responses to new symptoms seemed to be caused by the higher presence of comorbidities due to smoking, and perceptions of the current state of health. Individuals with a smoking history reported acting with urgency in seeking help and follow up, whereas patients who experienced low levels of concern were more easily reassured by clinicians, resulting in delays.

**Conclusions:**

Never and former smokers perceive, interpret, and respond to symptoms of lung cancer differently to smokers. However, few people attribute their lung symptoms to cancer initially, even with a smoking history. Interventions that drive increased urgency and vigilance in never smokers may be effective.

## 1 BACKGROUND

Lung cancer is the second most common cancer worldwide.^1^ While often caused by tobacco smoking, a significant minority of patients (approx 14%;^2^) have never smoked (hereafter referred to as ‘never smokers’). Lung cancer in never smokers can be considered a distinct disease from that in smokers, with unique molecular and biological characteristics.^3^

The relationship between smoking status, symptom appraisal and help‐seeking behaviour is complex. People who have never smoked are less likely to consider themselves at risk of lung cancer,^4^, ^5^, ^6^, ^7^ or worry about lung cancer.^8^ However, never smokers are more knowledgeable about both smoking‐related and non‐smoking related causes of cancer than smokers,^9^, ^10^ and are more likely to seek help for a lung cancer ‘alarm’ symptom than smokers.^11^ Delays in total time to diagnosis seem to be unaffected by smoking status, despite these differences.^12^, ^13^

Symptom prevalence and comorbid disease affect symptom appraisal and behaviour. Smokers frequently experience respiratory symptoms such as cough, breathlessness and tiredness,^14^ which may become normalised. People who smoke are more likely to have lung‐related comorbidities such as chronic obstructive pulmonary disease, known to increase delays in help‐seeking as patients or clinicians attribute the lung cancer symptoms to the pre‐existing disease.^13^, ^15^, ^16^ However, regular contact with health services to manage chronic disease can trigger opportunistic investigations, for example, changes in blood tests,^17^, ^18^ and lung cancer is often diagnosed incidentally, including through ‘non‐cancer’ pathways.^19^ Overall, much more is known about smokers' health behaviours than never smokers' health behaviours, particularly in the time between noticing a symptom and being diagnosed with cancer.^5^ More research is needed to understand the potential barriers and delays experienced by this growing group of patients, and how their needs differ from patients with a smoking history.

The Common Sense Model of Illness Self‐Regulation is a theoretical model which illustrates how individuals interpret and cope with symptoms or illness.^20^ The model links the perceived cause of the symptom, what the consequences might be, and whether behaviours (e.g. help‐seeking) will resolve the cause of the symptom or reduce negative emotions.^21^ Individuals regularly re‐appraise the symptom according to how they have reacted and what has happened so far. This model is particularly relevant to this study which investigates symptom interpretation, emotions and behaviours in relation to diagnosis of lung cancer, and provides a framework to explore the impact of smoking history. Other theoretical frameworks relating to symptom appraisal (e.g. the Symptom and illness attitude model; SIAM) do not conceptualise the link between symptoms and help‐seeking, and the longitudinal aspect of re‐appraisal.^22^

The aim of this study was to explore lung cancer patients' experiences of diagnosis to conceptualise similarities and differences by smoking history. The Common Sense Model of Illness Self‐Regulation was used after analysis to interpret the findings for this paper.

## 2 METHODS

### 2.1 Study design

Our qualitative interview study used semi‐structured telephone interviews with lung cancer patients across the UK. The interviews covered their experiences and perspectives in relation to lung cancer symptoms, risks and diagnosis. The study was considered and approved by the UCL Research Ethics Committee (project ID 17701/001).

### 2.2 Sampling and recruitment

#### 2.2.1 Patients

Individuals who received a lung cancer diagnosis in the previous 12 months were recruited by a specialist recruitment company (Taylor McKenzie Ltd; TM). Individuals were excluded if they had previously been diagnosed with lung cancer, but could be included if they had had other cancers in the past. TM invited individuals who previously consented to be contacted about research and reached out to support groups, charities and patient organisations through social media. To compare experiences by smoking history, participants included current smokers (*n* = 9), former smokers (*n* = 11) and people who never smoked (*n* = 20).

#### 2.2.2 Data collection

Participants were interviewed via telephone after providing verbal audio‐recorded consent. Semi‐structured interview discussion guides (see Appendix 1) were developed specifically to address the aims of the study, drafted by the study qualitative researchers (GB and SvO), and revised following feedback from patient representatives, academics and clinicians. Patient interviews explored patients' perceptions in relation to lung cancer risk and symptoms, their decision to visit primary care about their symptoms, and their experiences of the diagnostic pathway.

All interviews were conducted by SvO, a qualitative researcher experienced in health research who has no specialist clinical knowledge of lung cancer. Interviews were audio‐recorded and transcribed verbatim.

#### 2.2.3 Data analysis

Framework analysis was used to process the interview data.^13^ Initially all interview transcripts were read and summarised by SvO. 15 percent of interview transcripts were independently coded by a second qualitative researcher (GB). SvO and GB developed a coding framework based on emerging themes and topics covered in the interview guides. Both researchers then compiled separate tables for each patient group according to smoking status summarising data for each theme. All authors discussed these tables and identified/agreed on key themes in relation to the research aim. The results were further refined using the Common Sense Model of Illness Self‐Regulation.^14^ We used the model to consider how data portrayed participants' interpretation of their symptoms (called ‘threat’ in the model), what emotions they experienced, how they formulated ideas about what to do, and how they re‐appraised their symptoms as things progressed. The model was chosen after analysis, while considering how to best link the themes together.

In total 40 patients (Table 1) were interviewed.

**TABLE 1:** Patient demographics

	Current smoker (*n* = 9)	Never smoker (*n* = 20)	Former smoker (*n* = 11)
Sex			
Male	4	5	2
Female	5	15	9
Ethnicity			
White British	9	18	11
Asian British	0	2	0
Mean age	56.25	51.55	59.9
Range age	39–75	35–68	52–69
Significant physical comorbidity	6 (67%)	7 (35%)	5 (45%)
Average no. of symptoms at first presentation to primary care	1.7	1.2	1.2
Time since diagnosis			
Up to 3 months	3	4	2
3–6 months	0	10	2
6–9 months	1	2	3
9–12 months	2	3	3
Preferred not to say	3	1	1

## 3 FINDINGS

Many aspects of participants' diagnostic journeys did not differ according to smoking status. For example, participants in all groups had multiple investigations, periods of watchful waiting and a variety of sources of healthcare advice and consultation. Participants' appraisal, coping and re‐appraisal also showed some similarities. For example, very few participants initially suspected lung cancer. In this paper, we focus on the differences between the groups to capture what might be useful in developing tailored interventions to drive earlier diagnosis of lung cancer.

**FIGURE 1:**
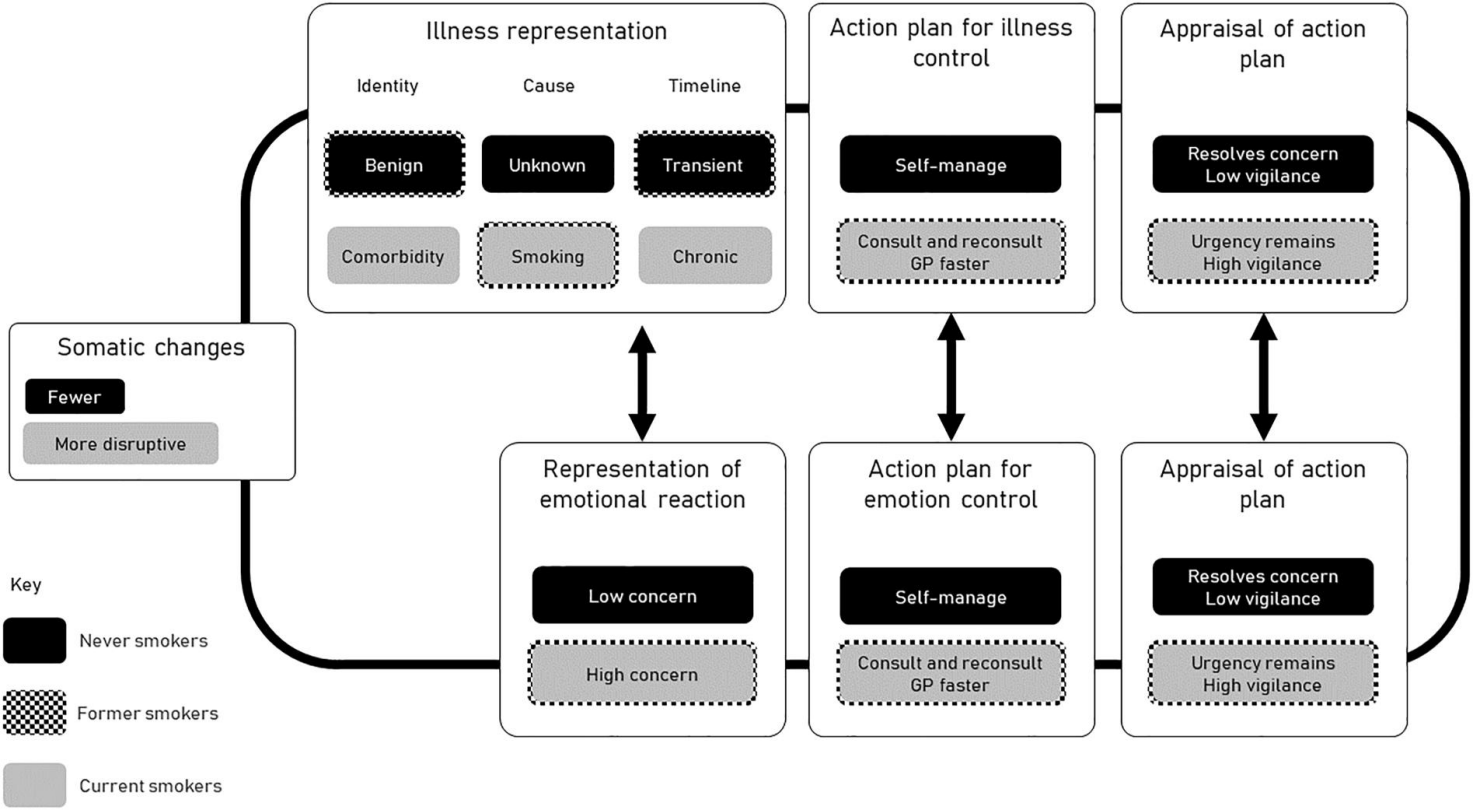
Summary of differences according to smoking status based on the common sense model of illness self‐regulation^23^

We have characterised these differences using the Common Sense Model of Illness Self‐Regulation (see Figure 1) Where former smokers show similarities with either smokers or never smokers, we indicate this with a dotted outline in the Figure.

### 3.1 Identity and timeline

The current smokers in our participant sample experienced dramatic somatic changes (e.g. lump in neck, coughing up blood). Some current smokers and former smokers experienced symptoms that triggered emergency healthcare such as collapse, or seizures. They also tended to have more than one symptom, whereas the never smokers in our sample mainly had a single or no symptoms in advance of their diagnosis.

Very few participants attributed their symptoms to lung cancer. Some current smokers had one or more chronic conditions (e.g. asthma, COPD) and often attributed their symptoms to exacerbations of these:

I just thought it was my COPD, with weather and it was like going cold and rainy, I just thought that they were like flu symptoms, something like that, but when it doesn’t go away, that’s when you start thinking, digging a bit deeper.(Participant 35, current smoker, female, 39 yrs old).

Never smokers described symptoms that were not particularly disruptive or invasive, and participants mainly attributed their symptoms to normal seasonal illness (e.g. a cold) or environmental factors such as hay fever or recent building work. They described how it was easy to dismiss their symptoms and several participants assumed at first that they did not require a visit to the GP.

Just a minor cough […] I first remember the cough, chesty cough starting in sort of August […] But then I’d come back, and we’d had…the ceiling had fallen down, I’d cleaned up all the plaster dust, and I just put it down to that.(Participant 5, never smoker, female, 42 yrs old)

Former smokers shared experiences with both current smokers and never smokers, but were more like never smokers in the way they interpreted their symptoms. Most participants in our sample attributed their symptoms to benign causes such as chest infections or a cold. However, they were also more aware of the potential risk of serious lung disease:

I had a few thoughts but cancer wasn’t one of them. It definitely wasn’t. I thought a chest infection. I was surprised when she said it was a flare up of my asthma, but I thought at least it’s not something I know can be controlled. When I saw the GP she thought it might be COPD and that was a scary thought. COPD at 52.(Participant 20, former smoker, female, 52 years old)

### 3.2 Causal link to smoking

We found that all participants were aware of the causal link between lung cancer and smoking. However, at the time of experiencing symptoms, participants had a variety of ways of considering the causal link between their symptoms and their smoking history.

While most former smokers and current smokers did not identify their symptoms as signs of lung cancer (as described in previous theme), many described an awareness of being at risk of cancer due to their smoking history. This affected some help‐seeking behaviours, for example, one participant reported that they wanted the doctor to *“double‐check”* their tests due to a concern about their smoking history. It also affected symptom appraisal; for example, one participant recognised that a new cough was important, due to her risk as a long term smoker. For these participants, lung cancer was not the most prominent identity in their minds, but the causal link to smoking was present.

However, many participants with a smoking history did not perceive a causal link between their symptoms and smoking, due to being in good health. One participant did not perceive a causal link between her symptoms and smoking, because they were not the classic symptoms associated with lung cancer:

I've never wheezed, or anything like that, I've never coughed, I've never had a cough with this cancer or anything.(Participant 17, current smoker, female, 47 yrs old)

Similarly, many never smokers felt healthy and well, and attributed their symptoms to mild illness or an unknown cause. The interviews in this study were conducted during the COVID‐19 pandemic, and many patients attributed low level respiratory symptoms to this initially. Mainly, this group did not consider that they could be at risk of lung cancer due to the strong association with smoking.

I would associate lung cancer with smoking. And even when my voice started getting hoarse I remember talking to someone at work and she said, “Mine does that sometimes,” so I just dismissed it. But I berate my sheer stupidity now that I didn’t even think it was something beyond that.(Participant 4, never smoker, female, 53 yrs old).

Former smokers in our sample reported mixed causal attributions to their symptoms. Some had respiratory comorbidities such as asthma and COPD, whereas others strongly identified as active, healthy individuals. Despite regularly smoking for a long time in the past, one participant did not attribute her symptoms to lung cancer because of her age:

I just thought that I’m young, I’m too young to have lung cancer because I was 54, I just thought old people got it.(Participant 36, former smoker, female, 54 yrs old)

### 3.3 Emotional reaction to symptoms: Level of concern

Emotional reactions to symptoms were dependent on the context of investigations. For example, three participants had their cancers diagnosed following routine investigations for other conditions, and had no current symptoms. For symptomatic patients, the severity of their symptoms and their own perceptions of risk strongly influenced their emotional reaction. Current smokers and former smokers described feeling quite worried, particularly in relation to dramatic symptoms such as coughing up blood or lumps in the neck.

I had a friend that died of cancer not that long before hand, and he found a lump on his neck, and so I thought, “This could be a bit dodgy”.(Participant 17, current smoker, female, 47 yrs old)

In contrast, some never smokers had low levels of concern due to the fact that they attributed their symptoms to benign, self‐limiting attributions which were not worrisome:

Yeah, because if you have a cough and then you have a cold, but it's just a cough and it's not going away. That was before COVID‐19, so yeah, it's like a cough that's not going away, then the family history, but you try and push it away. You really don't think it's gonna be serious, so you try to not think about it too much.(Participant 12, never smoker, male, 46 yrs old)

Some former smokers had reactions more like current smokers, with a higher level of overall concern. Others described low levels of concern, pushing the symptoms to the back of their minds or experiencing “denial”:

I kept thinking well, I feel fine, I've got no real symptoms, I'm healthy and fit. The symptoms are minor, annoying, that was all. So I think it was a bit, I was in this sort of disbelief probably, and sort of denial that anything was happening.(Participant 16, former smoker, male, 55 yrs old)

Thus, the perception of risk related to smoking seemed a minor factor driving emotional responses compared to the severity of the symptom and causal explanation.

### 3.4 Action plans for emotional and illness control

Participants coped with their interpretations and concerns about their symptoms differently, with noticeable differences in experience according to smoking status. Smokers and former smokers described urgency in seeking help from primary care, or reconsulting when their symptoms did not resolve.

They had done a chest x‐ray, they had done blood tests, and they decided, as I say, that it was a chest infection. The same on the second occasion, the third occasion, as I say, they put it down to pneumonia.[…] I pushed my doctor and said that I still had problems with my chest and concerns, and he offered me more antibiotics or another x‐ray(Participant 32, current smoker, male, 59 years old)

Most smoker participants maintained a high degree of vigilance until they were diagnosed. One participant described telephoning the hospital weekly until he received an appointment.

I was phoning weekly to Dr [X]’s secretary to see what's happening […] I got eventually, on the sixth of May, I got an appointment with the respiratory doctor at [hospital], and that's when he told me I had a tumour and it was cancerous (Participant 1, current smoker, male, 75 yrs old)

Several patients from all three groups were prescribed antibiotics and other medications to try and clear their symptoms. Current and former smokers reported that they re‐appraised their symptoms as ‘not resolved’, and reconsulted with the GP as a coping strategy fairly quickly, particularly when prescribed medication did not work.

I had two lots of antibiotics so I went back probably about a week and a half, two weeks after I had the first set of antibiotics and he gave me some stronger antibiotics the second time and then probably it seemed to sort of clear up a bit. (Participant 36, former smoker, female, 54 yrs old).

In contrast, we interpreted that never smokers were fairly reassured by GP advice relating to a benign disease because it was in line with their own symptom interpretation. This led to long delays before reconsulting, sometimes for as long as several months. This meant that participants were often reassured by clinicians that there was no immediate need to progress investigations:

She said okay we’ll give you these antibiotics. Took those, didn’t do any good. And so I remember speaking to her again and her saying well the next stage would usually be an x‐ray but we’re trying to keep people out of hospital. But I remember saying you know, it’s not debilitating, it’s just something that’s a bit weird and doesn’t seem to be going away.(Participant 23, never smoker, male, 48 years old)

Such was the low level of concern by never smokers, that they were easily reassured by the doctor even in the presence of serious symptoms:

She said, “Don’t worry unless you’re coughing up more than a teaspoon of blood,” which is quite surreal really. That was her statement. So I thought, well that doesn’t seem right, but because she was a GP I just took it at face value.(Participant 4, never smoker, female, 53 yrs old).

Due to the initial low level of concern, never smokers' re‐appraisal of symptoms showed a pattern of feeling resolved and maintaining low vigilance. For example, delays in receiving healthcare were not perceived as worrisome:

I was never given any reason to be concerned by the medics, I guess, especially since the narrative they were, sort of, putting around it seemed to be much more infection/menopausal, cough clinic. And then with the spirometry test that reassured me that it didn't need urgent attention. So that when the delay came through COVID it was more frustrating but I didn't ever, ever think it would be critical.(Participant 38, never smoker, female, 52 yrs old).

Overall, participants' re‐appraisal of their coping strategies was strongly related to the response they had received from healthcare professionals. Participants who were swiftly sent for investigations had their concern validated. Current and former smokers whose concerns were not resolved maintained a high level of vigilance to ensure their symptoms continued to be investigated. In contrast, never smokers were often reassured easily leading to a feeling of resolution and low vigilance.

## 4 DISCUSSION

This is the first study to explicitly compare never smokers, former smokers and current smokers in terms of experience of symptoms, help‐seeking and management in primary care. Qualitative comparison integrating the Common Sense Model of Illness Self‐Regulation has revealed novel information about interpretation of symptoms, personal risk of lung cancer and patient behaviour in primary care according to smoking status.^14^ Comorbidities due to smoking, and perceptions of the *current state of health* are driving interpretation and coping responses to new symptoms for current smokers, rather than perceived lung cancer risk. This novel finding builds on several qualitative studies that also found a lower self‐perception of risk in never smokers.^5^, ^7^, ^14^

Our results show that multiple factors drove delays in help‐seeking for patients who had never smoked. For example, having symptoms which were not particularly dramatic or disruptive, which is known to affect cancer symptom interpretation.^24^ This provoked low levels of concern about their symptoms, and delays in help‐seeking and reconsultation behaviour. In contrast, previous studies noted that current smokers were more likely to delay help‐seeking than never smokers.^11^, ^14^ This may be a difference caused by our lung cancer patient sample, rather than symptomatic patients in the community who have different intentions and behaviours.^11^, ^14^

We found that symptom appraisal and help‐seeking of patients in the former smoker group is more complex to understand (and therefore predict), as they shared some similarities in experience with both current and never smokers. This mirrors survey research showing that former smokers perceive their risk of lung cancer as lower than current smokers,^25^ yet have some similarities in symptom appraisal with current smokers.^26^

A key message from our research is that perceived urgency is an important driver of timely help‐seeking and follow up, and that patients who experience low levels of concern are less likely to remain vigilant to symptoms. Building on our rapid review of evidence, this study fills a gap in evidence around the way that never smokers seek help for respiratory symptoms, and re‐appraise their symptoms following contact with health services.^5^

### 4.1 Study limitations

One study limitation is the lack of ethnic minority participants, and a younger sample on average than the lung cancer population. This was caused by difficulties with recruitment during the pandemic, despite efforts to recruit through specialist agencies and with incentives to participate. We also recruited more women than men to our study, which is a significant limitation and one which may have influenced our findings. However, this is the first study of its kind to explore differences in lung cancer symptom appraisal and behaviour according to smoking history, and demonstrated the utility of a model which links symptom interpretation to emotional and behavioural responses.

Given that our study was conducted during the COVID‐19 pandemic, many participants' experiences of symptom appraisal and help‐seeking may have been affected in a way that restricts generalisability. Recent research has highlighted that lung cancer control has been particularly affected in comparison to other cancer types, due to the impact of messaging around non‐urgent medical care, the overlap in symptoms between COVID‐19 and lung cancer, and the reliance on telephone consultation in primary care.^27^, ^28^, ^29^ Additionally, our remote approach to data collection may have influenced what participants were willing to share with the researcher, and the researcher could not pick up on body language and gesture. Finally, some participants were interviewed more than 6 months after their diagnosis, so they may have forgotten certain details and had more time to reflect and interpret their experiences.

### 4.2 Clinical implications

Managing non‐specific symptoms such as coughs presents a significant challenge for primary care clinicians. Interventions such as safety netting (offering advice about the likely duration of symptoms and when to reconsult) are seen as best practice in this scenario.^30^, ^31^, ^32^ However, clinicians are likely to be less urgent or specific in their advice if they perceive the patient to be at lower risk (e.g. for those with no smoking history).^33^, ^34^ Our results suggest that inviting urgency and vigilance is a crucial part of the safety netting encounter in order to prompt timely reconsultation. Some clinicians report that they do not want to provoke anxiety for their patients.^34^, ^35^ Therefore optimal communication practices should be developed that encourage urgency and vigilance without unnecessarily causing worry.

## 5 CONCLUSIONS

Timely diagnosis of lung cancer is extremely challenging for all patients, due to the non‐specific nature of commonly experienced symptoms. While patients with a smoking history continue to be at higher risk of lung cancer than those without, lung cancer in never‐smokers represents a growing proportion of patients. Never and former smokers perceive, interpret, and respond to symptoms of lung cancer differently to smokers and the common sense model of illness self‐regulation is likely to help underpin the development of tailored interventions. For example, based on our evidence related to symptom identity and emotional responses to symptoms, interventions that drive increased urgency and vigilance in never smokers may be effective.

## CONFLICT OF INTEREST

The authors declare no conflict of interest.

## Data Availability

Research data are not shared.

## APPENDIX 1 INTERVIEW TOPIC GUIDE

Patient Experience of symptoms, help‐seeking and risk factors in lung cancer.

This document is intended to be a guide. The following topics/questions/prompts are not exhaustive and the researcher may probe or follow the participants’ line of interest as is appropriate for the purpose of this study.

### INTRODUCTION TO STUDY

- Introduce self and briefly explain project.
- Provide all potential participants with a copy of participant information sheet
- Answer any questions and explain process.
- Remind participants of their rights, including confidentiality, right to withdraw, etc.

After full explanation of project has been given, if participant says they will take part:

- Ask participant to read and sign participant consent form.
- Participants in telephone interviews will be asked to verbally confirm they agree to each of the statements on the consent form. Their verbal consent will be audio‐recorded.

### INTERVIEW

Structured questions to map the diagnostic pathway.

1. Which signs/symptoms did you experience before you went to the GP?
2. How did the symptoms develop over time?
3. When did you first see the GP about these symptoms?
4. How did your GP visit go?
  Prompts
  - What happened during the visit?
  - Did they do any investigations?
  - Did they arrange any tests?
  - Did you go back for further visits?
  - How many visits?
  - Over how long a period?
  - Did you see any other healthcare professionals?
5. Who diagnosed you?
  Prompts
  - When did you receive diagnosis?
  - Who diagnosed you?
  - Where were you diagnosed?
  - What happened after your diagnosis?

Semi‐structured interview questions to explore symptom experience and interpretation, help‐seeking, illness schemas, experience of primary and secondary diagnostic and care pathways, and social and life history prior to diagnosis. The interview discussion guide will include the below topics, but will not be finalised until after phase 1 (evidence synthesis) has been completed.

### INTERVIEW TOPICS

1. Could you tell me a bit more about the signs or symptoms you were experiencing?
2. When did you first notice changes?
  Prompts
  ‐ What made you notice?
  ‐ What was happening at the time?
  ‐ Who noticed them?
  ‐ At that time, what did you think was causing it?
  ‐ Have your thoughts about this changed over time?
  ‐ If so, how?
  ‐ Did you look up any information about the symptoms?
    ▪ Internet / family / friends / health care professional
  ‐ Did you talk to anyone else about the symptom?
    ▪ How did you feel about telling others about your symptoms? **Stigma**
    ▪ Do you think it’s a common symptom?
  ‐ What did you family/friends think about your symptoms? **Stigma**
    ▪ Other people?
    ▪ Did they encourage you to seek help?
    ▪ Did you trust their judgements?
3. How did you react the first time you noticed the symptom(s)? Move to question 5 if GP is mentioned.
4. What made you decide to visit the GP?
5. How long was it before you decided to see the GP/make an appointment?
  Prompts
  - What influenced your decision?
  - Were there any personal reasons that kept you from going to see the GP? **Stigma**
  - Would it have affected other people if you went to the GP? Would anyone have said anything to you?
  - Some people say they feel a bit embarrassed, would you be like that? **Stigma**
  - Some people worry they are wasting GP time, would you be like that?
6. Was it easy/simple to see the GP?
  - If not, why?
  Prompts
  - Difficult to get to?
  - Interfere with work/other priorities?
  - Getting an appointment?
7. How would barriers that were mentioned be overcome?
  Prompts
  - Is this something that someone else could have helped you with?
  - Why them? How could they have helped?
  - Would it be made easier if something were changed in your community?
8. How did that first GP visit go?
  Prompts
  - What happened?
  - How did you feel during the visit?
  - And after?
  - Were you concerns dealt with/what was the outcome of the visit?
  - What did you understand about the symptoms after the visit?
9. What happened next?
  Prompts
  - Probe on experiences in relation to further visits to GP (did they see the same GP, or not? Why/why not?).
  - Were you referred? (probe who referred them, why, to whom were they referred, experiences, how long did it take, etc.)
  - Who diagnosed you in the end? (probe experiences about the diagnosis, how long after first visit, what did they say, did you understand what was said, how did you feel, did you talk to family/friends? Did you receive support? Etc).
10. How soon after diagnosis did you start treatment?
  Prompts
  - Were there delays?
    - What were they caused by?
    - Do you think this delay has had a negative impact on you?
    - What could have prevented the delay?
11. How is the treatment going?

### CLOSE AND DEBRIEF

- Thank you
- Explain what will be done with information
- Remind where contact details can be found
- Check participant is happy and answer any questions
